# Fangyukangsuan granules ameliorate hyperuricemia and modulate gut microbiota in rats

**DOI:** 10.3389/fimmu.2024.1362642

**Published:** 2024-04-30

**Authors:** Qing-zheng Zhang, Ji-rui Zhang, Xue Li, Jin-long Yin, Li-ming Jin, Zhuo-ran Xun, Hao Xue, Wan-qi Yang, Hua Zhang, Jingyong Qu, Zhi-kai Xing, Xu-min Wang

**Affiliations:** ^1^ College of Life Sciences, Yantai University, Yantai, Shandong, China; ^2^ Jilin Ginseng Academy, Changchun University of Chinese Medicine, Changchun, Jilin, China; ^3^ Department of Food Science and Engineering, Jilin Business and Technology College, Changchun, Jilin, China; ^4^ Key Laboratory of Biotechnology and Bioresources Utilization, Dalian Minzu University, Dalian, China

**Keywords:** hyperuricemia, Fangyukangsuan granules, gut microbiota, molecular docking, xanthine oxidase

## Abstract

Hyperuricaemia (HUA) is a metabolic disorder characterised by high blood uric acid (UA) levels; moreover, HUA severity is closely related to the gut microbiota. HUA is also a risk factor for renal damage, diabetes, hypertension, and dyslipidaemia; however, current treatments are associated with detrimental side effects. Alternatively, Fangyukangsuan granules are a natural product with UA-reducing properties. To examine their efficacy in HUA, the binding of small molecules in Fangyukangsuan granules to xanthine oxidase (XOD), a key factor in UA metabolism, was investigated via molecular simulation, and the effects of oral Fangyukangsuan granule administration on serum biochemical indices and intestinal microorganisms in HUA-model rats were examined. Overall, 24 small molecules in Fangyukangsuan granules could bind to XOD. Serum UA, creatinine, blood urea nitrogen, and XOD levels were decreased in rats treated with Fangyukangsuan granules compared to those in untreated HUA-model rats. Moreover, Fangyukangsuan granules restored the intestinal microbial structure in HUA-model rats. Functional analysis of the gut microbiota revealed decreased amino acid biosynthesis and increased fermentation of pyruvate into short-chain fatty acids in Fangyukangsuan granule-treated rats. Together, these findings demonstrate that Fangyukangsuan granules have anti-hyperuricaemic and regulatory effects on the gut microbiota and may be a therapeutic candidate for HUA.

## Introduction

Hyperuricemia (HUA) is a metabolic disorder characterised by high blood levels of uric acid (UA), the main product of purine metabolism (1). In the absence of uricase, which converts urate into water-soluble allantoin, the resulting UA is not efficiently excreted (2). The equilibrium of UA homeostasis relies on its synthesis in the liver and elimination through the kidneys and intestines. Elevated intake of foods high in purines (such as seafood, beer, and red meat), long-term consumption of fructose, and unhealthy body status under excessive stress conditions can disrupt UA homeostasis, resulting in elevated plasma UA levels (3, 4). High blood UA levels may induce excessive deposition of UA crystals in the body, which can cause gout (5). HUA is also a risk factor for renal damage, diabetes, hypertension, and dyslipidaemia (6, 7). Medications commonly used to treat HUA can be categorised as uricosuric agents (e.g., probenecid and benzbromarone) or xanthine oxidase (XOD) inhibitors (e.g., allopurinol) (8). However, the pharmacological interactions of probenecid and the potential for benzbromarone-associated hepatotoxicity remain unclear (9, 10). Moreover, allopurinol is also associated with severe side effects including skin rashes, allergic reactions, and gastrointestinal toxicity (11, 12). Therefore, naturally active substances that can effectively regulate UA levels and prevent HUA with minimal adverse effects are required.

The gut microbiota participates in the maintenance and maturation of the immune system, metabolism, and other processes to ensure intestinal barrier stability and a balanced intestinal environment (13–16). Disturbances in the gut microbiota can increase the risk of many diseases such as obesity, type 2 diabetes, non-alcoholic liver disease, cardiometabolic disease, and malnutrition (17). The gut microbiota participates in the metabolism of purines and UA (18–20). Several studies have revealed altered gut microbiota in patients with gout; for example, *Bacteroides caccae* and *Bacteroides xylanisolvens* were abundant in the gut of patients with HUA, whereas *Faecalibacterium prausnitzii* and *Bifidobacterium pseudocatenulatum* were absent. Gut microbial remediation has minimal side effects in humans and is widely accepted as a cost-effective treatment option. Zhou et al. (21) revealed that chlorogenic acid can improve the symptoms of HUA by increasing the relative abundance of short-chain fatty acid-producing bacteria, including *Bacteroides*, *Prevotellaceae* UGC-001, and *Butyricimonas* in the gut of mice, and by adjusting the purine and glutamate metabolism of the gut microbiota. Chicory intervention in high-UA quail models alleviates HUA by increasing the abundance of probiotic bacteria (*Bifidobacterium* and Erysipelaceae), reducing the abundance of pathogenic bacteria (*Helicobacteriaceae*), inhibiting the lipopolysaccharide/Toll-like receptor 4 (TLR4) axis inflammatory response, and significantly decreasing serum UA levels (22). Curcumin treatment can inhibit the overgrowth of opportunistic pathogens, including *Escherichia/Shigella* and *Bacteroides*, in UA nephropathy and increase the relative abundance of bacteria such as *Lactobacillus* and *Ruminococcaceae*, which can produce short-chain fatty acids (23).

Fangyukangsuan granules contain celery (*Pimpinella brachycarpa* (Kom.) Nakai), corn silk, and *Chlorella pyrenoidosa*. Celery is rich in beneficial compounds including carbohydrates, proteins, dietary fibres, polyphenols, flavonoids, and minerals. Celery can reduce swelling and diuresis, lower blood sugar levels and blood pressure, alleviate oxidative stress, and has anti-tumour properties (24–26). Celery is widely used in traditional medicine as a congener of medicine and food (27, 28). Corn silk, a part of the female flower (stigma) of corn plants, is an established Chinese herbal medicine. Corn silk contains various compounds including flavonoids, isorhamnetin, phenolics, alkaloids, and tannins (29, 30). Corn silk possesses various health benefits, such as disease prophylaxis including gout, rheumatism, rheumatoid arthritis, and urinary ailment prevention, and exhibits antitumour, hypoglycaemic, antioxidant, and antifungal activities (31–33). Several *in vivo* and clinical studies have reported that corn silk is safe for human consumption (31–34). *C. pyrenoidosa*, one of the earliest commercially developed algal species, is often used as a dietary supplement because it is a rich source of protein as well as pigments, fatty acids, growth factors, vitamins, and minerals (35). *C. pyrenoidosa* has been reported to exhibit various biological activities including immunomodulatory, antioxidant, antidiabetic, antihypertensive, and antihyperlipidaemic activities (36, 37). Functionally, *C. pyrenoidosa* helps clear heat and dampness, celery significantly reduces UA and relieves pain, and corn silk promotes diuresis. Thus, the combination of these three ingredients helps to degrade UA crystals, increases urine volume, and facilitates the excretion of UA, thereby reducing UA levels.

In the present study, we investigated the effects and possible mechanisms of action of Fangyukangsuan granules on HUA. The HUA rat model was used to confirm that Fangyukangsuan granules could decrease UA levels *in vivo*, and the underlying mechanism was explored with regard to changes in the gut microbiota. This study lays the foundation for the development of natural substances to prevent or ameliorate HUA, and provides an effective treatment strategy for patients with HUA.

## Materials and methods

### XOD molecular docking and inhibition

To explore the multitarget effects of the active ingredients in plant molecules, XOD, a key factor in UA metabolism, was selected as the receptor protein for molecular docking experiments. Three types of small-molecule plant compounds were identified by searching a database of traditional Chinese medicines. Repeated small molecules were not deleted to distinguish the sources of the components. The dimensional crystal structure of XOD (ID: 1N5X) was obtained from the Protein Data Bank (http://www.rcsb.org). Discovery Studio 2019 was used to analyse the XOD structures, incorporate ligands, remove water molecules, delete the B-chain, and add polar hydrogen molecules (38). Discovery Studio was also used to dock small ligand molecules with XOD proteins. The TEI docking scores were considered as thresholds, and the binding sites of the top three small-molecule compounds with regard to the molecular docking scores and the proligands of the XOD receptor proteins were analysed. Solutions of XOD and xanthine were mixed with solutions of different concentrations at ambient temperature, and the inhibition of XOD was measured by the absorbance at 290 nm.

### Chemicals and reagents

Hypoxanthine (99%), potassium oxonate (97%), and sodium carboxymethylcellulose (CMC–Na) were acquired from Shanghai Macklin Biochemical Co., Ltd. (Shanghai, China). Allopurinol was purchased from Shanghai Yuanye Bio-Technology Co., Ltd. (Shanghai, China). Serum UA, blood urea nitrogen (BUN), creatinine (Cr), and XOD levels were assessed using kits purchased from the Nanjing Jian Cheng Bioengineering Institute (Nanjing, China).

### Animals and experimental design

Forty-two specific pathogen-free male Sprague-Dawley rats (weight, 300 ± 3.2 g) were purchased from Jinan Pengyue Experimental Animal Breeding Co., Ltd. (Jinan, China). The animals were maintained under standard feeding conditions (24°C, 12-h light/dark cycle) with free access to food and water. After 7 days of adaptation, the rats were randomly assigned to seven groups: blank control (CON), model (MOD), allopurinol-treated (AP), Fangyukangsuan granule-treated (QY), celery-treated (QC), corn silk-treated (YM), and *Chlorella*-treated (QZ), with six rats per group; rats were housed with others in the same group. Except for the rats in the CON group, which were intragastrically administered equal volumes of aqueous 0.5% CMC–Na, the other groups were intraperitoneally injected with 300 mg/kg potassium oxonate dissolved in a 0.5% CMC–Na solution and intragastrically administered 500 mg/kg hypoxanthine for 8 weeks. After 4 weeks, rats in the AP group received 15 mg/kg allopurinol via oral gavage once daily; those in the QY group were administered 0.9 g/kg Fangyukangsuan granules via gavage; and those in other groups (QC, YM, and QZ groups) received 0.9 g/kg celery powder, corn silk powder, or *Chlorella* powder, respectively, via gavage for 4 weeks. All procedures involving animals were carried out following the Yantai University guidelines for the Care and Use of Laboratory Animals. The Yantai University Animal Ethics committee provided ethical clearance for the experiments involving animals, under the permit number 20220619003.

### Measurement of biochemical indices

On the last day of the experiment, eye socket blood samples were collected and serum was extracted by centrifugation (10 min, 4°C, 3500 rpm), and then preserved at −80°C for biochemical assays. Following the manufacturer’s instructions, commercially available kits were used to measure the serum UA, BUN, Cr, and XOD levels.

### Haematoxylin and eosin staining

Rat kidneys and liver were fixed for 36 h at 4°C in 4% paraformaldehyde overnight and embedded in paraffin. Each specimen was cut into 5-μm thick slices. The tissue slices were de-paraffinised in xylene and rehydrated in a series of ethanol solutions. The sections were dehydrated using graded ethanol and xylene solutions after H&E staining. Organizational structure of the tissue was observed under 200X magnification.

### Metagenomic sequencing

Metagenomic sequencing was conducted on rat faeces collected on the final day of the animal study. Total DNA was extracted using a kit according to the manufacturer’s instructions and the purity and integrity of the extracted DNA were assessed using 1% agarose gel electrophoresis. An Illumina HiSeq 2500 platform (San Diego, CA, USA) was used to sequence the qualified library preparations and paired-end reads were produced for each sample. Raw read quality checks were performed using KneadData, which also eliminated host reads (39). The rat (mRatBN7.2) reference database was downloaded, and Bowtie2 contained in KneadData was used with Bowtie2 options “–very-sensitive –dovetail” for read mapping to the human genome to eliminate human sequences. Trimmomatic in KneadData was employed with the trimmomatic options “SLIDINGWINDOW:4:20 MINLEN:50” to perform read trimming. The metagenomic phylogenetic analysis tool MetaPhlAn4 was used to profile the microbial community composition at the species level based on quality-filtered reads (40). For metagenomic taxonomic profiling, MetaPhlAn4 integrates reference sequences from isolates and metagenome-assembled genomes and can significantly enhance the metagenomic mapping of gut microbiomes. All taxonomic information on the microorganisms was reported as relative abundance. To analyse the metabolic function (gene family and pathway) of the microbial community, the HMP Unified Metabolic Analysis Network (HUMAnN3) was employed (41). Pathways were annotated using MetaCyc IDs. Additionally, the pathway abundance reads per kilobase values were normalised to relative abundance using the humann_renorm_table function.

### Statistical analysis

All statistical analyses were performed using R (v4.03). Alpha and beta diversities were assessed using species-level taxonomic and metabolic pathway data. Alpha diversity was estimated using the Shannon index. Nonmetric multidimensional scaling (NMDS) analysis was performed to visualise sample relationships across different groups using the metaMDS function of the vegan package. The dissimilarity matrix was used to conduct statistical comparisons for beta-diversity measures, employing permutational multivariate analysis of variance (ANOVA) and comparing the distance between groups using the Wilcoxon test. Stacked bar plots of phylum abundance were visualised using ggplot2 v3.4.0 in the R project; pheatmap v1.0.12 was used to construct relative abundance plots of the 30 most abundant species. STAMP was used to identify differentially abundant metabolic pathways and species (42). Sankey plots were constructed through their origins.

## Results

### Detection of binding interactions between Fangyukangsuan granule components and XOD

**Figure 1:**
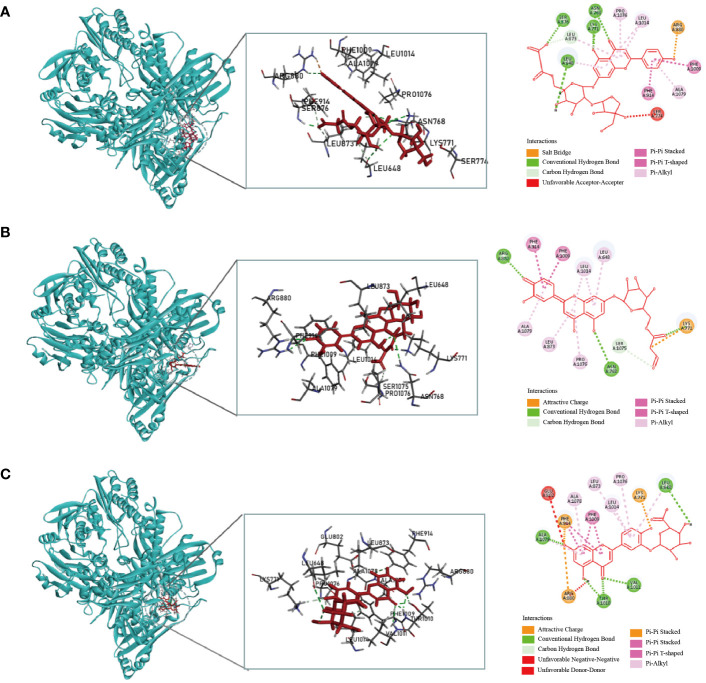
Molecular docking simulation of the malonylapiin **(A)**, luteolin 7-O-(6′′-malonylglucoside) **(B)**, and luteolin-3-O-beta-D-glucuronide **(C)** peptides with XOD.

Considering the docking scores of the primary ligand, TEI, as thresholds, 24 small-molecule compounds with scores higher than this threshold were selected (**Table 1**). Among these, 50% were derived from celery, 33% from corn silk, and 16% from *C. pyrenoidosa*. **Figure 1** shows the LibDock scores of the top three small molecules that bind to XOD. Malonylapiin formed four conventional hydrogen bonds and one carbon–hydrogen bond with XOD and electrostatically interacted with Arg880 in chain A and hydrophobically with Leu1014, Pro1076, and Ala1079 in chain A. Luteolin 7-O-(6′′-malonyl glucoside) formed two conventional hydrogen bonds and one C–H bond with XOD and showed electrostatic interaction with Arg415 in chain A and hydrophobic interactions with Leu648, Leu1014, Leu873, Pro1076, and Ala1079 in chain A. Luteolin-3-O-β-D glucuronide formed four conventional hydrogen bonds with XOD and showed electrostatic interactions with Lys771, Phe914, and Arg880 in chain A.

**Table 1:** The results of the molecular docking between Fangyukangsuan granules and XOD.

Identification	Source	Element Composition	Score
Malonylapiin	Celery	C_29_H_30_O_17_	163.775
Luteolin 7-O-(6’’-malonylglucoside)	Celery	C_24_H_22_O_14_	152.882
Luteolin-3-O-beta-D-glucuronide	Celery	C_21_H_18_O_12_	151.357
Luteolin-3-O-beta-D-glucuronide	Celery	C_30_H_26_O_13_	146.094
Apiin	Celery	C_26_H_28_O_14_	143.586
Robinin	Corn silk	C_33_H_40_O_19_	142.766
Luteolin-7-(2-O-Apiosylglucoside)	Celery	C_26_H_28_O_15_	142.425
Luteolin-7-O-6’- acetylglucoside	Celery	C_23_H_22_O_12_	139.674
4H-1-Benzopyran-4-one, 2-(3,4-dihydroxyphenyl)- 6-b-D-glucopyranosyl-5,7-dihydroxy	Corn silk	C_21_H_20_O_11_	135.768
Octacosane	Corn silk	C_28_H_58_	134.795
Docosanoic acid	Corn silk	C_22_H_44_O_2_	133.725
Hexacosane	Corn silk	C_26_H_54_	133.148
Apigenin 7-O-glucoside	Celery	C_21_H_20_O_10_	132.945
Chrysoeriol	Corn silk	C_16_H_12_O_6_	132.634
Thermopsoside	Celery	C_22_H_22_O_11_	132.563
Isorhamnetin	Corn silk	C_16_H_12_O_7_	130.935
Luteolin	Celery	C_15_H_10_O_6_	129.543
Diosmetin-7-O-beta-D-glucoside	Celery	C_28_H_32_O_15_	128.687
Methyl 19-methyleicosanoate	*Chlorella pyrenoidosa*	C_22_H_44_O_2_	126.892
5,8,11,14-Icosatetraynoic acid	*Chlorella pyrenoidosa*	C_20_H_24_O_2_	126.694
Chlorogenic acid	Celery	C_16_H_18_O_9_	126.417
α-Linolenic acid	*Chlorella pyrenoidosa*	C_18_H_30_O_2_	125.924
Docosanoic acid	Corn silk	C_22_H_44_O_2_	124.879
γ-Linolenic acid	*Chlorella pyrenoidosa*	C_18_H_30_O_2_	124.671
TEI	Proligand	C_16_H_16_N_2_O_3_S	124.36

**Figure 2:**
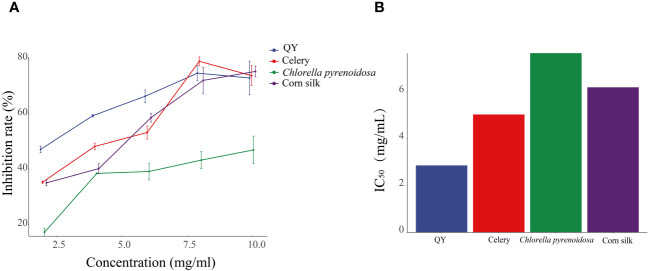
Inhibitory effects of QY, celery, corn silk, and *C. pyrenoidosa* on XOD activity. **(A)** Inhibition rate. **(B)** IC_50_ values.

As the concentration increases, the inhibition of XOD activity by QY, celery, corn silk, and *C. pyrenoidosa* gradually intensifies (**Figure 2**). At a concentration of 8 mg/mL, the QY group reached its peak inhibition at 75.71%, with an IC_50_ value of 2.86 mg/mL. In comparison, the inhibition rates of XOD by Chlorella and corn silk were lower, with IC_50_ values of 7.66 mg/mL and 6.20 mg/mL, respectively. Overall, QY exhibits a superior inhibitory capability against XOD compared to the individual components.

### Effect of Fangyukangsuan granules on the biochemical indices of HUA rats

**Figure 3:**
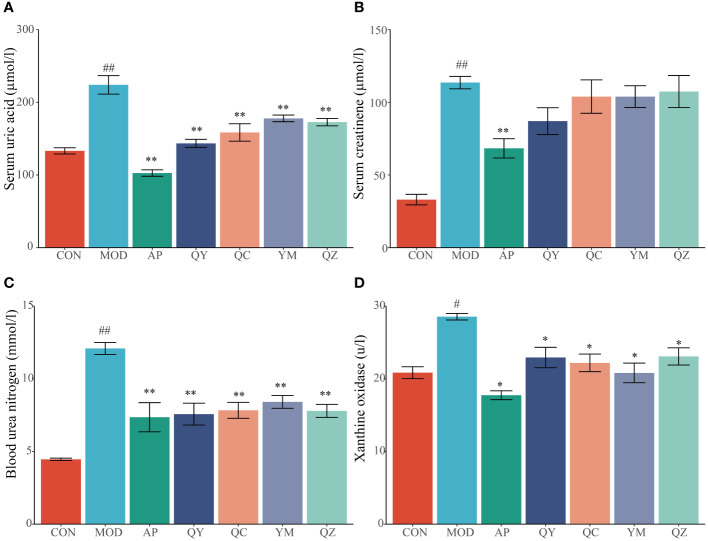
Effects on biochemical indicators related to hyperuricemia. **(A)** Serum uric acid. **(B)** Serum creatinine. **(C)** Blood urea nitrogen. **(D)** Xanthine oxidase. Compared with the MOD group, *p < 0.05, **p < 0.01; Compared with the CON group, ^#^p < 0.05, ^##^p < 0.01.

After 28 days, rats fed hypoxanthine and potassium oxonate showed significant changes in serum UA levels (**Figure 3A**). Compared with that in the CON group, the level of serum UA in the MOD group increased significantly (p < 0.01), indicating the success of the model. Conversely, the serum UA levels in the AP, QY, QC, YM, and QZ groups were significantly lower (p < 0.01) than that in the MOD group. The level of serum Cr in the MOD group was significantly (p < 0.01) higher than that in the CON group (**Figure 3B**). The reduction in serum Cr level was significant (p < 0.01) only in the AP group compared with that in the MOD group (**Figure 3B**). The level of serum BUN level in the MOD group was significantly higher (p < 0.01) than that in the CON group whereas those in the AP, QY, QC, YM, and QZ groups were significantly (p < 0.01) reduced (**Figure 3C**). The serum XOD level in the MOD group was significantly (p < 0.05) higher than that in the CON group whereas serum BUN levels in the AP, QY, QC, YM, and QZ groups were significantly (p < 0.05) reduced (**Figure 3D**).

### Effects of Fangyukangsuan granules on the liver and kidney in rats

**Figure 4:**
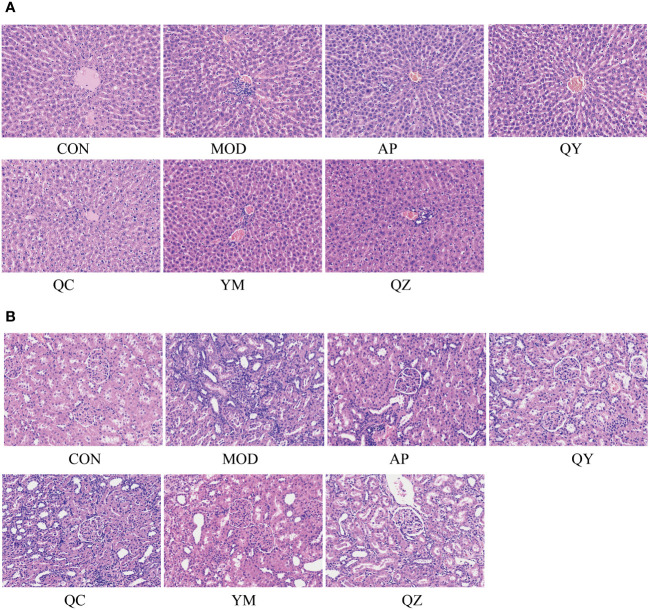
The micrographs of kidney and liver sections were stained with H&E and visualised at 200X magnification. **(A)** Liver. **(B)** Kidney.

The morphological characteristics of hepatocytes in the CON group were normal (**Figure 4**). In the MOD group, the hepatic cord structure appeared unclear, with evident signs of hepatocyte vacuolar degeneration, swelling, and water degeneration. Following treatment, the hepatic cord structure became clearer and hepatocyte vacuolisation was reduced. Compared to that in the CON group, H&E staining in the MOD group revealed several visible histological changes, including cytoplasmic vacuolisation and dilation, renal tubule swelling, proximal tubule necrosis, and indistinct boundaries between adjacent proximal tubule cells. However, compared with those in the MOD group, rats in the administration group showed improvements in renal tubules and glomerulopathy, with a relatively clear cytoplasm, reduced tubule swelling, obvious boundaries between adjacent proximal tubules, and less proximal tubule necrosis.

### Effects of Fangyukangsuan granules on gut microbiota in the HUA rat model

**Figure 5:**
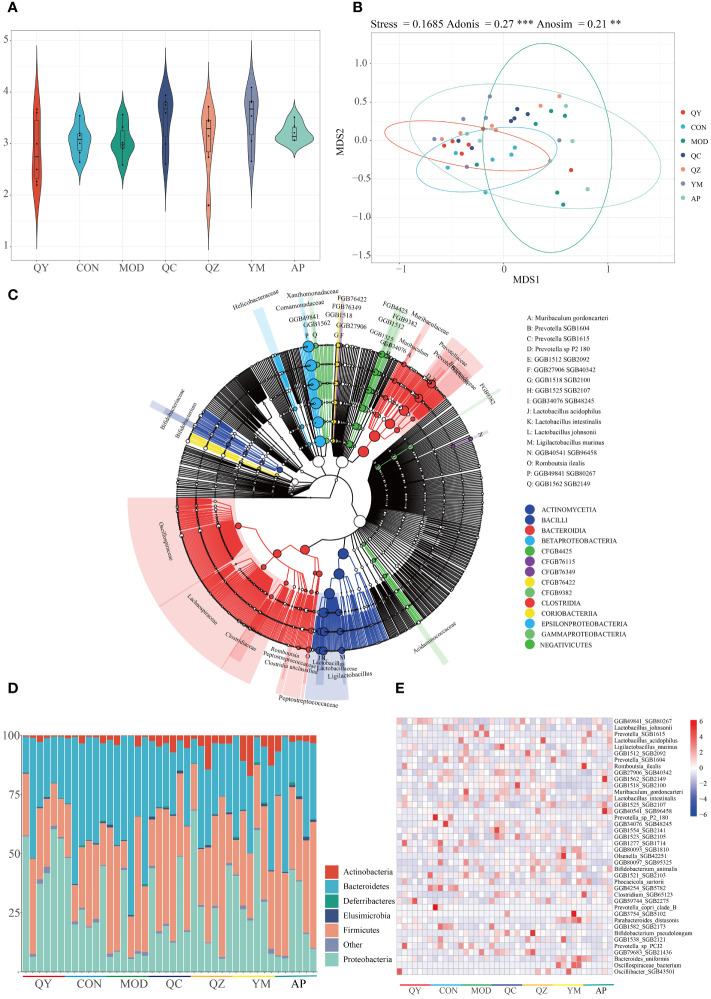
Diversity of gut microbiota among groups. **(A)** Shannon index. **(B)** Non-metric multidimensional scaling of faeces microbiota. **(C)** Phylogenetic trees of microbiota at the species level. **(D)** Relative abundances of gut microbiota at the phylum level. **(E)** Heat map of relative abundances of bacteria at the species level.

Metagenomic sequencing provided insights regarding alterations in the gut microbiota of treated rats. Initially, we examined the Shannon index to determine microbiota diversity. The Shannon index was lower in the MOD group than in the CON group. The Shannon index increased in the QY group (**Figure 5A**). NMDS suggested that the structure of the gut microbiota of rats in the QY group was closest to that of rats in the CON group (**Figure 5B**). Betaproteobacteria and Bacilli were the most abundant at the class level, with Bacteroidia, Bacilli, and Clostridia being the most diverse in the annotated results among all samples (**Figure 5C**).

At the phylum level (**Figure 5D**), the relative abundances of Bacteroidetes and Firmicutes were markedly increased in the HUA group compared to those in the CON group. QC and AP administration attenuated HUA-induced reduction in the abundance of Proteobacteria and increased that of Bacteroidetes. At the species level, 692 taxa were identified and the top 40 taxa were visualised using a heatmap (**Figure 5E**); 20 of these taxa were Bacteroidetes, and 16 were Firmicutes. Eight *Prevotellaceae* were Bacteroidales, and eight *Lactobacillaceae* were Lactobacillales. Six of the 40 most abundant species were *Prevotella* and *Lactobacillus*. Compared with that in the MOD and AP groups, the relative abundance of *Prevotella* SGB1615 was elevated in the other groups. The MOD, QZ, and AP groups maintained a higher relative abundance of *Lactobacillus acidophilus*. The MOD, QZ, and QC groups had higher relative abundances of *Muribaculum gordoncarteri*, whereas the AP and QC groups had higher relative abundances of *Ligilactobacillus murinus*. Other species exhibited varying degrees of change among the different groups.

**Figure 6:**
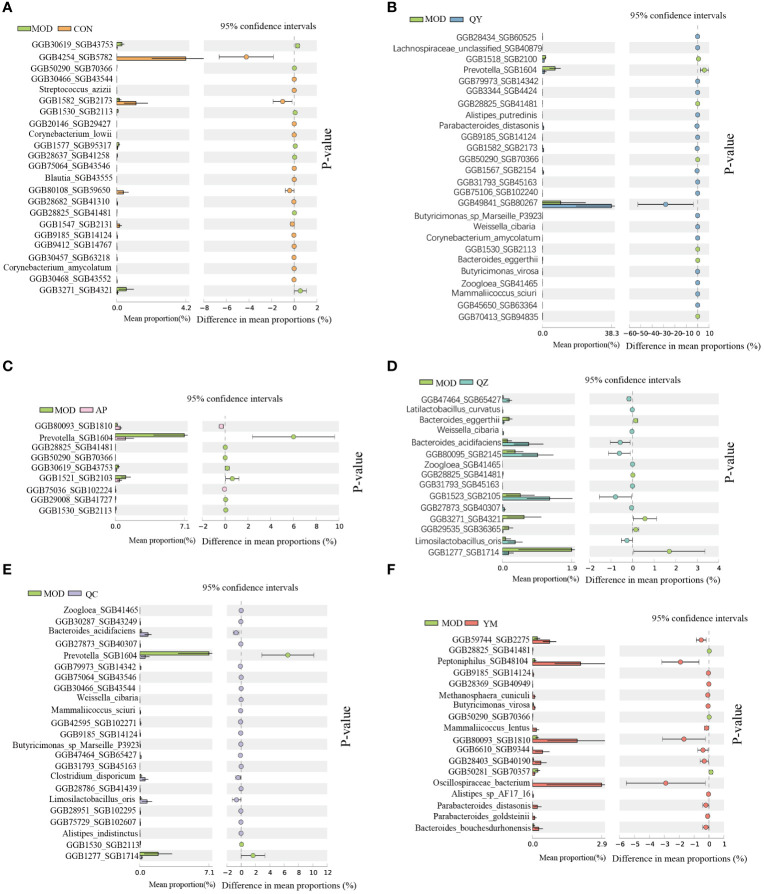
Comparative taxonomic characteristics of different groups with MOD at the species level. Analysis was performed using STAMP. The q‐values are based on Welsh’s t-test with Benjamin–Hochberg FDR correction (q value < 0.05). **(A)** CON group. **(B)** QY group. **(C)** AP group. **(D)** QZ group. **(E)** QC group. **(F)** YM group.

We further analysed the significant differences in bacterial abundance at the species level to determine the similarities and differences between the different groups (**Figure 6**). Compared to that in the MOD group, the QY group contained 26 taxa, including six Bacteroidales and two *Butyricimonas*; the AP group contained nine taxa; and the CON group contained 23 taxa that exhibited significant changes in abundance. *Prevotella* SGB1604 showed high abundance in the MOD group, from which the abundances in the QY, AP, and QC groups differed significantly. Compared to that in the MOD group, significant increases in the abundance of *Corynebacterium amycolatum* were observed in the QY and CON groups, that of *Mammaliicoccus sciuri* in the QY and QC groups, and that of *Parabacteroides distasonis* and *Limosilactobacillus oris* in the QZ and QC groups.

### Effects of Fangyukangsuan granules on gut microbial metabolism

**Figure 7:**
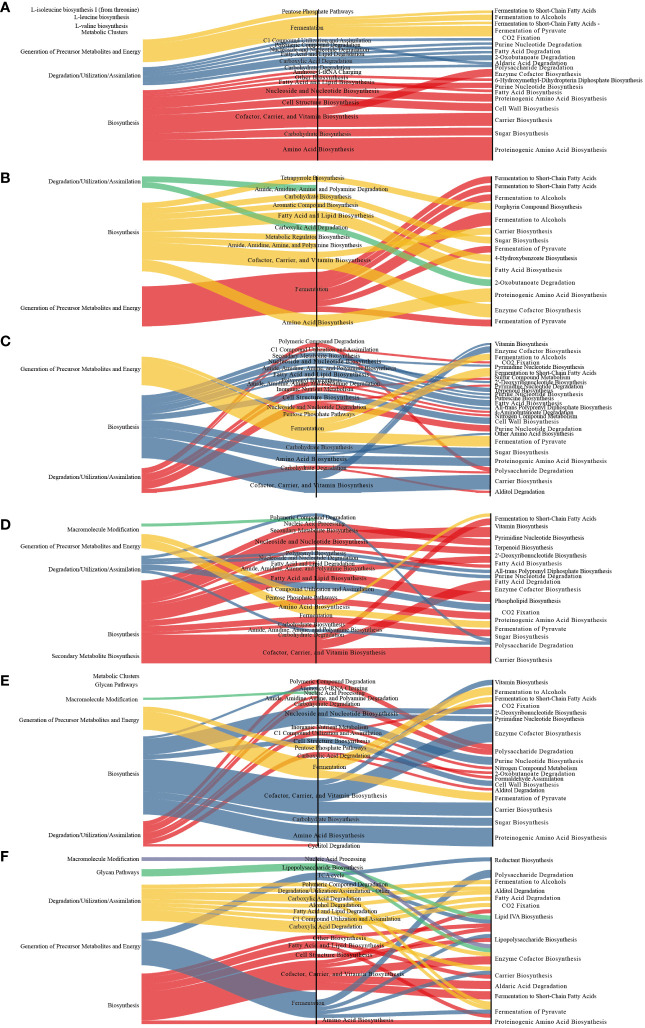
Sankey diagram displaying the associations between the differential functional pathways and metabolic reactions. The functional pathways are significantly different between different groups and the MOD group. Metabolic reactions were grouped according to the MetaCyc pathway categories. **(A)** QY group. **(B)** CON group. **(C)** QC group. **(D)** QZ group. **(E)** YM group. **(F)** AP group.

Compared to those in the MOD group, several metabolic pathways were significantly altered in the other groups. The differential metabolic pathways are primarily involved in biosynthesis and the generation of precursor metabolites and energy. Fermentation pathways were the main differential metabolic pathways altered in the latter category whereas cofactor, carrier, and vitamin biosynthesis were the main altered biosynthesis-related differential metabolic pathways (**Figure 7**).

**Figure 8:**
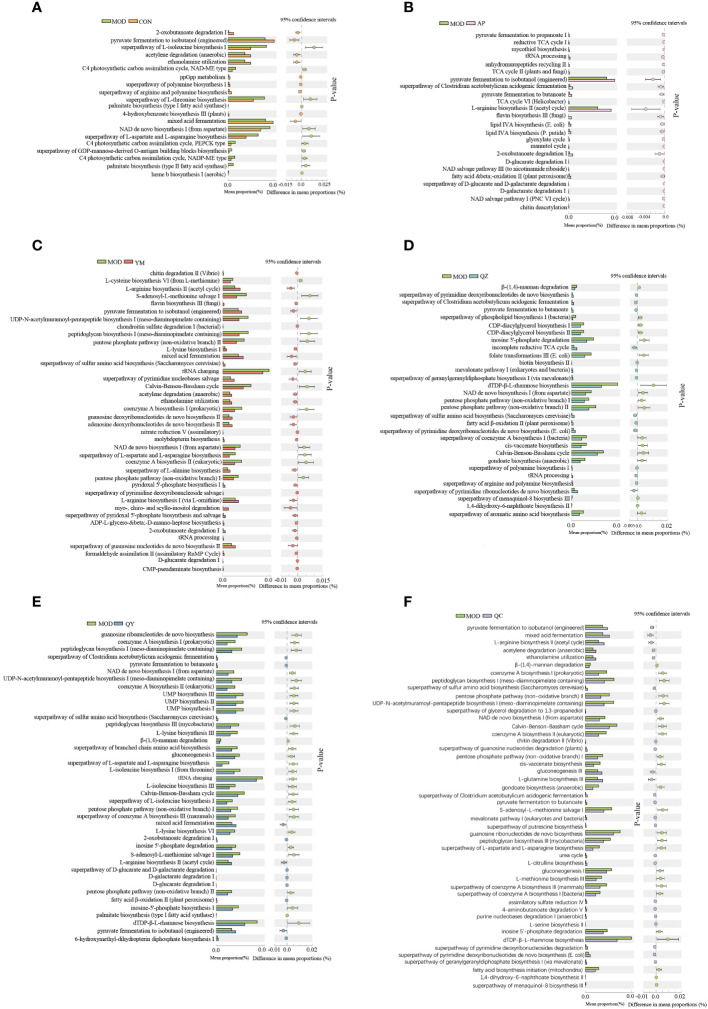
Comparative taxonomic characteristics of different groups and the MOD group according to the functional path. Analysis was performed using STAMP. The q‐values are based on Welsh’s t-test with Benjamin–Hochberg FDR correction (q value < 0.05). **(A)** CON group. **(B)** AP group. **(C)** YM group. **(D)** QZ group. **(E)** QY group. **(F)** QC group.

Compared to those in the CON group, the abundances of nine different metabolic pathways were significantly reduced in the MOD group (**Figure 8**). Compared with those in the MOD group, 40 distinguishable taxa were altered in the QY group. Pyruvate fermentation to isobutanol (engineered) significantly increased in all groups except the QZ group. NAD *de novo* biosynthesis I (from aspartate) significantly decreased in all groups, except in the AP group. Ten pathways showed significant differences across four experimental groups: L-arginine biosynthesis II (acetyl cycle), superpathway of L-aspartate and L-asparagine biosynthesis, Calvin–Benson–Bassham cycle, pyruvate fermentation to butanoate, mixed acid fermentation, pentose phosphate pathway (non-oxidative branch) I, 2-oxobutanoate degradation I, superpathway of *Clostridium acetobutylicum* acidogenic fermentation, pentose phosphate pathway (non-oxidative branch) II, and superpathway of sulphur amino acid biosynthesis (*Saccharomyces cerevisiae*). Nine pathways exhibited significant differences between the AP and QY groups. Functional analysis of the gut microbiota revealed decreased amino acid biosynthesis and increased fermentation of pyruvate to short-chain fatty acids in the QY group.

## Discussion

HUA remains a cause of global health problems associated with changes in lifestyle and dietary patterns, such as excessive intake of high purine-content foods, excessive levels of stress and anxiety, and unhealthy physical status (4, 43). HUA has been linked to hypertension, cardiovascular disease, diabetes, and metabolic syndromes (6, 44). Long-term maintenance of HUA may cause sodium urate crystallisation in the kidneys and joints, which can lead to gout, a painful condition characterised by recurrent inflammation (45) that can seriously threaten the quality of life of patients. Therefore, the regulation of UA metabolism using natural herbal medicines is attracting increasing attention. Zhao et al. (46) revealed that ripened Pu-erh tea can significantly lower serum UA levels and that modulation of amino acid metabolism may be the primary mechanism underlying the antihyperuricaemic effect induced by this tea. Withaferin A, isolated from *Withania somnifera*, significantly prevented renal fibrosis and improved kidney function by decreasing UA levels via the regulation of XOD and transporter genes in renal tubular cells (47). Another study showed that *Rhizoma smilacis glabrae* extract reduced hepatic XOD activity in a dose-dependent manner and ameliorated inflammatory cell infiltration, tubular dilation, and vacuole formation in renal tubular cells (48). Siwu decoction, a classical prescription of traditional Chinese medicine, exerts antihyperuricaemic and anti-inflammatory effects by inhibiting hepatic XOD activity, regulating renal organic ion transporter expression, and suppressing renal NLRP3 inflammasome activation (49). Thus, the use of natural herbal medicines with UA-lowering abilities to improve kidney injury may be a promising strategy for treating HUA.

Fangyukangsuan granules are composed of celery, corn silk, and *C. pyrenoidosa*. Celery and corn silk reduce blood UA levels and alleviate HUA-induced kidney damage (50). XOD is a key enzyme involved in the metabolism of hypoxanthine and xanthine during UA production (51, 52). In this study, we used molecular docking, a technique to predict structural interactions between a ligand and receptor, to reveal that 26 small molecules in Fangyukangsuan granules could bind to XOD with higher docking scores than those of the original ligands. Most of these small molecules were derived from celery or corn silk. These substances interact with various XOD amino acid residues, such as Thr1010, Glu802, Leu1014, Leu873, Val1011, Phe649, Arg880, and Phe914, which are believed to affect the active centre of XOD (53). Furthermore, our results showed that Fangyukangsuan granules significantly reduced serum XOD levels in the HUA rat model. Thus, Fangyukangsuan granules exerted a significant inhibitory effect on XOD and constitute a promising and effective natural XOD inhibitor.

Serum UA levels comprise the main marker for HUA research. In this study, elevated serum UA levels in the HUA group compared to those in the CON group validated the establishment of the HUA rat model. Notably, both Fangyukangsuan granules and their components significantly decreased UA levels in the HUA rat model, with Fangyukangsuan granules having a greater effect on serum UA reduction than any single component. As an XOD inhibitor, AP effectively reduces serum UA levels by inhibiting UA synthesis in the liver. In HUA rat models treated with AP, serum UA and XOD levels were significantly decreased, verifying that AP utilised a similar mechanism of action in this model. Furthermore, Fangyukangsuan granules showed an effectiveness similar to that of AP, demonstrating promising value in HUA treatment. Kidney damage can lead to reduced clearance rates as well as elevated blood concentrations of Cr and BUN, along with increased production of proinflammatory cytokines and chemokines (54). Fangyukangsuan granules decreased Cr and BUN levels in the HUA rat model. Histopathological H&E micrographs showed a similar trend, indicating that the administration of Fangyukangsuan granules effectively mitigated liver and kidney injuries in the HUA rat model.

Notably, the intestines also play a crucial role in the elimination of UA from the body. Endogenous UA from the blood is secreted directly into the gut lumen through urate transporters in all gut segments (55, 56). This process relies on the maintenance of gut homeostasis by the gut mucosal barrier (57). The gut microbiota can regulate intestinal barrier stability and the balance of the intestinal environment (16); alternatively, imbalances in the gut microbiota can disturb UA metabolism, leading to increased serum UA levels. Therefore, gut microbiota may be a potential target for alleviating HUA (54). In this study, we aimed to investigate the effect of Fangyukangsuan granules on the gut microbiota under HUA conditions. We demonstrated that Fangyukangsuan granules significantly reshaped and restored the composition of the gut microbiota in HUA-model rats. The species composition of the gut microbiota in rats treated with Fangyukangsuan granules was similar to that in healthy rats. Consistent with previous results (58), an increased relative abundance of Bacteroidetes was observed in HUA-model rats. At the species level, the abundances of *Weissella cibaria*, *Butyricimonas virosa*, *Parabacteroides distasonis*, and *Alistipes putredinis* were significantly increased compared with those in the HUA group. *W. cibaria* can enter the digestive system and form colonies, enhancing natural killer cell activity and immunological functions (59). The major end-products of *B. virosa* are butyric and isobutyric acids, together with smaller amounts of acetic, propionic, and succinic acids (60). According to Koh et al. (61), *P. distasonis* exhibits anti-inflammatory and anti-cancer activities. These effects are likely caused by the inhibition of TLR4 and Akt signalling as well as the promotion of apoptosis. *A. putredinis* may be linked to the microbial metabolic pathways of gluconeogenesis, fatty acid oxidation, palmitoleate biosynthesis, and folate conversion (62). Functional analysis of the gut microbiota showed that amino acid biosynthesis decreased in Fangyukangsuan granule-treated rats. Notably, UA is an important metabolite involved in amino acid biosynthesis. Moreover, fermentation of pyruvate to short-chain fatty acids increased in Fangyukangsuan granule-treated rats. These metabolites are produced by the microbial fermentation of undigested fibres and help maintain epithelial integrity and promote immunological tolerance (63). Thus, Fangyukangsuan granules may regulate intestinal immune homeostasis through gut microbiota in HUA-model rats. However, further studies are required to investigate the specific mechanisms underlying these effects.

In summary, this study revealed that Fangyukangsuan granules possess a strong ability to lower UA levels and effectively downregulate serum biochemical indices. We also demonstrated the potential of Fangyukangsuan granules in repairing organ damage and reducing inflammatory responses in an HUA rat model. From the perspective of the gut microbiota, Fangyukangsuan granules exerted potential effects that supported the recovery of gut microbial community structure and increased the number of beneficial bacteria. Intervention in amino acid biosynthesis and the fermentation of pyruvate to short-chain fatty acids may be the major mechanisms underlying the antihyperuricaemic effects of Fangyukangsuan granules. Together, these findings suggested that Fangyukangsuan granules represent promising therapeutic candidate for HUA.

## Data availability statement

The datasets generated for this study can be found in the National Genomics Data 430 Center: https://ngdc.cncb.ac.cn/search/?dbId=&q=CRA013747.

## Ethics statement

The animal study was approved by The Ethics Review Committee of Yantai University. The study was conducted in accordance with the local legislation and institutional requirements.

## Author contributions

QZ: Formal analysis, Software, Writing – original draft, Writing – review & editing. JZ: Data curation, Formal analysis, Methodology, Writing – review & editing. XL: Formal analysis, Software, Writing – review & editing. JY: Data curation, Formal analysis, Methodology, Writing – review & editing. LJ: Data curation, Formal analysis, Writing – review & editing. ZX: Investigation, Software, Writing – review & editing. HX: Conceptualization, Data curation, Writing – review & editing. WY: Conceptualization, Data curation, Writing – review & editing. HZ: Investigation, Software, Writing – review & editing. JQ: Conceptualization, Supervision, Validation, Writing – review & editing. KX: Conceptualization, Funding acquisition, Supervision, Validation, Writing – review & editing. XW: Conceptualization, Funding acquisition, Methodology, Supervision, Writing – review & editing.
